# Sensor Driven Resource Optimization Framework for Intelligent Fog Enabled IoHT Systems

**DOI:** 10.3390/s26010348

**Published:** 2026-01-05

**Authors:** Salman Khan, Ibrar Ali Shah, Woong-Kee Loh, Javed Ali Khan, Alexios Mylonas, Nikolaos Pitropakis

**Affiliations:** 1 School of Computing, Gachon University, Seongnam 13120, Republic of Korea; drkhan@gachon.ac.kr; 2 Department of Computer Software Engineering, University of Engineering & Technology, Mardan 23200, Pakistan; ibrar@uetmardan.edu.pk; 3 Cybersecurity and Computing Systems Research Group, Department of Computer Science, University of Hertfordshire, Hatfield AL10 9AB, UK; j.a.khan@herts.ac.uk (J.A.K.); a.mylonas@herts.ac.uk (A.M.); 4 Cybersecurity and Computer Science, School of Science and Technology, The American College of Greece, 6 Gravias Str., Aghia Paraskevi, 153 42 Athens, Greece

**Keywords:** fog computing, resource allocation, healthcare, real-time applications

## Abstract

Fog computing has revolutionized the world by providing its services close to the user premises, which results in reducing the communication latency for many real-time applications. This communication latency has been a major constraint in cloud computing and ultimately causes user dissatisfaction due to slow response time. Many real-time applications like smart transportation, smart healthcare systems, smart cities, smart farming, video surveillance, and virtual and augmented reality are delay-sensitive real-time applications and require quick response times. The response delay in certain critical healthcare applications might cause serious loss to health patients. Therefore, by leveraging fog computing, a substantial portion of healthcare-related computational tasks can be offloaded to nearby fog nodes. This localized processing significantly reduces latency and enhances system availability, making it particularly advantageous for time-sensitive and mission-critical healthcare applications. Due to close proximity to end users, fog computing is considered to be the most suitable computing platform for real-time applications. However, fog devices are resource constrained and require proper resource management techniques for efficient resource utilization. This study presents an optimized resource allocation and scheduling framework for delay-sensitive healthcare applications using a Modified Particle Swarm Optimization (MPSO) algorithm. Using the iFogSim toolkit, the proposed technique was evaluated for many extensive simulations to obtain the desired results in terms of system response time, cost of execution and execution time. Experimental results demonstrate that the MPSO-based method reduces makespan by up to 8% and execution cost by up to 3% compared to existing metaheuristic algorithms, highlighting its effectiveness in enhancing overall fog computing performance for healthcare systems.

## 1. Introduction

Cloud computing provides computation, storage and network services to the customers over a wide geographical network. However, the centralized architecture and higher magnitude of user requests not only result in higher bandwidth consumption but also an increase in latency as well. This increase in service delivery or communication latency is due to the centralized nature and multi-hop distance between core data centers and end users. Real-time applications like smart manufacturing, smart transportation and smart healthcare require immediate response time. However, the cloud is unable to provide quick response time, which is unacceptable to real-time delay-sensitive applications [1]. Therefore, there is an immense need to resolve the above issue and provide the cloud resource at the edge of the network to avoid delay.

Fog computing is an extension of cloud computing where the resources are provided very close to the edge network to reduce the delay and network traffic. The basic aim is to minimize the latency and energy consumption and increase the throughput. The sudden increase in IoT devices and the data they generate for computation makes it more complicated. It is estimated that the growing number of devices connected to the Internet globally will generate 73 zettabytes of data by 2025 [2]. The increasing demand in user requests has compelled academia and industry to explore and develop potential solutions for real-time applications. Under the given circumstances, fog computing is the most suitable solution. However, fog devices are resource constrained. To effectively handle incoming user requests, there is a critical need for efficient resource allocation, task scheduling, and load balancing mechanisms. These are essential to maximize the utilization of fog resources and minimize application latency, particularly for time-sensitive healthcare applications [3], which is of great importance. We contextualize our work within recent advances in system resilience, including moving target defense strategies based on game theory and Proactive Internet of Things (PIoT) defense frameworks. These approaches demonstrate the benefits of dynamic, adaptive mechanisms for reducing attack surfaces and enhancing IoT system reliability, highlighting the importance of proactive security in distributed network environments.

This study presents the novel resource management technique using Modified Particle Swarm Optimization to tackle the aforementioned challenges associated with the fog computing paradigm. The proposed technique is based on efficient resource allocation and task scheduling by optimizing system performance parameters in terms of system response time, execution cost and time. The contributions made to this paper are as follows:

- Proposing a three-tier architectural framework integrating cloud, fog, and IoT layers is a strategic approach for delivering reliable, low-latency, and intelligent healthcare services.
- An optimal resource allocation and task scheduling strategy is proposed using a Modified Particle Swarm Optimization technique for delay-sensitive healthcare applications. The proposed method enhances the resource utilization of fog devices while minimizing makespan and execution cost as key parameters.
- Analyzed and evaluated the proposed technique through several experimental iterations to ensure the effectiveness of the proposed technique in a given scenario.

The remainder of the paper is organized in the following sections: Section 2 overviews the relevant literature and identifies the current gaps in the context of this work. Section 3 presents the proposed three-tier architectural framework model. Section 4 illustrates the resource management and scheduling issue in fog computing. Section 5 exhibits the system design and optimization modeling. Section 6 discusses the experimental settings and simulation setup and reports the obtained results with respect to the existing state-of-the-art work. Finally, Section 7 concludes the research work and highlights the future work.

## 2. Related Work

Efficient resource management has emerged as one of the most critical concerns in the evolving landscape of modern computing. The ability to effectively allocate, monitor, and optimize computational resources directly influences the performance, scalability and responsiveness of any computing environment, be it cloud, fog or edge systems. As computing infrastructures continue to expand in complexity and heterogeneity, intelligent resource management mechanisms have become indispensable for achieving seamless service delivery, minimizing latency and maximizing utilization across distributed architectures [4]. The processing element in cloud computing is powerful enough to entertain high-demand requests, so resource allocation and scheduling are not big issues; rather, communication latency between core data centers and end-user devices creates a big and consistent problem. This issue brings academicians and industries to come up with a new computing paradigm known as fog computing. Fog computing brings cloud services close to the edge network; the services included are computation, storage and network services [5]. However, resource allocation and scheduling are rather difficult for resource-constrained devices. Therefore, there is a need for proper optimization techniques to utilize maximum fog resources and reduce the overhead delay for real-time IoT applications [6]. Waleed et al. [7] propose a resource allocation strategy for 5G networks using the particle swarm optimization technique. The authors proposed a general version of the PSO algorithm that shows the PSO characteristics. However, the article lacks in terms of determining the architectural framework for the concerned 5G network and real-time application scenarios.

Alqahtani et al. [8] proposed a novel scheduling and load balancing approach for delay-sensitive applications. The proposed approach allocates resources and balances the load to optimize resources and load variance. Potu et al. [9] proposed a meta-heuristic algorithm for optimal resource allocation and scheduling to minimize the makespan and completion time. Abdulredha et al. [10] proposed a scheduling algorithm for bag-of-task in fog computing. The proposed approach optimizes the makespan and execution cost for optimal performance. Babar et al. [11] proposed an intelligent computation offloading algorithm for scalable edge computing. The proposed approach reduces the latency and improves the quality of service (QoS) as performance parameters. Zhang et al. [12] proposed a resource allocation strategy and trust computation-based blockchain framework in edge computing. The primary goal of the proposed approach is to provide authenticity and verification for search data. An optimal resource allocation and load balancing technique was developed for a fog computing environment targeting critical healthcare applications. The main aim of the proposed technique optimizes the metrics like latency, energy and network bandwidth [13]. Ahmad et al. [14] proposed a scalable and flexible multi-task orchestration architecture to efficiently manage heterogeneous resources in distributed computing environments. The proposed approach is a mapping and allocating of tasks for non-technical users that are relevant to IoT enterprises to minimize the round trip time. Ahmad et al. [15] proposed a real-time effort for formal job verification through optimal threshold value. The concerned approach is used for task monitoring and evaluation and reduces the overall CPU utilization, power consumption and response time as performance metrics. Jamil et al. [16] proposed a job scheduling algorithm for critical healthcare applications to minimize the average delay and energy consumption. Baburao et al. [17] proposed dynamic resource allocation to handle the load and optimize the system performance, like response time, network and latency. Wadhwa and Aron in [18] proposed a resource allocation technique to ensure maximum utilization of resources in a fog computing environment. The primary goal of the concerned approach is to reduce the execution time, delay, energy and network usage. Kaur and Aron in [19] proposed a hybrid meta-heuristics load balancing technique for scientific workflows. The concerned technique balances the workload and reduces the execution time. Similarly, recent work grows the importance of deep reinforcement learning (DRL) for task offloading and resource allocation in a three-tier computing framework to optimize multiple QoS objectives through RL [20,21]. Frameworks such as ReinFog show practical distributed implementations of DRL across fog and cloud [22]. Furthermore, DQN-based task allocation has been used to adaptively distribute workloads under dynamic cloud-fog-IoT network environments [23].

It is obvious from the above discussion that resource management is an important research domain to be applied in any computing paradigm. However, resource allocation, scheduling, load balancing, reliability and scalability are some of the major and important characteristic features in the resource management layer that enhance the overall system performance by using some optimization methods. However, most of the developed studies or applied techniques did not consider the real-time applications to integrate with the computing paradigm. Therefore, we propose a novel multi-level task execution algorithm using resource allocation and task scheduling for real-time application. The primary aim of the proposed technique is to reduce the makespan and execution cost of the computational nodes and enhance the overall system performance.

## 3. Proposed Architectural Framework Model

**Figure 1:**
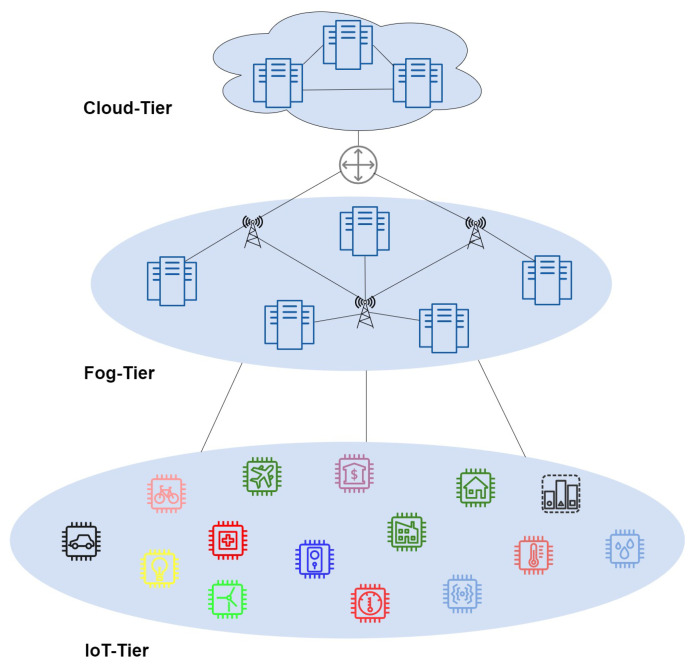
Overall fog computing architecture.

A three-layer hierarchical architecture integrating IoT, fog, and cloud layers is designed, as illustrated in Figure 1, to enable efficient coordination and resource management across the computing continuum.

### 3.1. Cloud Tier

The cloud layer constitutes the highest and most resource-intensive tier within the fog computing hierarchy. It comprises large-scale, centralized data centers equipped with extensive computational, storage and analytical capabilities. This tier is primarily responsible for handling complex, latency-tolerant and data-intensive operations that exceed the processing capacities of edge and fog layers, thereby ensuring global coordination, long-term data management and large-scale service orchestration across the distributed computing continuum. These data centers are centrally controlled but geographically distributed across a wide network. As a result, real-time requests must travel multiple hops to reach their destination, requiring rapid response times upon arrival [24]. This distance creates communication latency between the user request and response from the cloud server, which is unacceptable to the real-time IoT applications [25].

### 3.2. Fog Tier

The fog layer plays a crucial role in the multi-layer architectural framework, where there is a significant communication latency frequently produced by cloud computing due to its widely geographically distributed end points and multi-hop distance. Therefore, real-time needs an immediate response time for complex problems. The efficient utilization of fog resources needs proper resource utilization, which is only possible through an optimization technique.

### 3.3. IoT Tier

The IoT tier forms the lowest layer in the fog computing architecture and comprises sensors, actuators, and smart devices. These sensors include medical, temperature, light, humidity, aerial, and pollution sensors. IoT devices generate real-time requests and transmit them to nearby fog nodes for prompt processing, helping to minimize latency [26]. However, fog-based IoT facilitates end-to-end service delivery with rapid response times and improved quality of service (QoS) [27] and provides better and secure monitoring services [28] for real-time IoT applications [29].

## 4. Efficient Resource Allocation and Scheduling Problem in Fog Computing

Resource management plays a pivotal role in the efficiency and effectiveness of any computing paradigm. It encompasses several key components, including the following:

*Resource Allocation:* Refers to the dynamic provisioning of computational resources such as CPU cycles, memory, storage and network bandwidth according to task-specific demands and predefined system policies to achieve optimal utilization.

*Load Balancing:* It involves the equitable distribution of computational workloads across available nodes to mitigate bottlenecks, avoid resource overloading and sustain overall system efficiency and responsiveness.

*Task Scheduling:* Entails determining the optimal execution location and timing for each task, whether processed locally or offloaded to fog, edge or cloud servers. This decision is guided by multiple factors, including latency constraints, energy efficiency and the current state of resource availability.

*Reliability:* It ensures the robustness and continuity of system operations by maintaining fault tolerance, consistency and service availability, even under component failures or network disruptions.

*Scalability:* It represents the system’s capability to accommodate increasing workloads and expanding infrastructure while maintaining performance stability and functional integrity.

**Figure 2:**
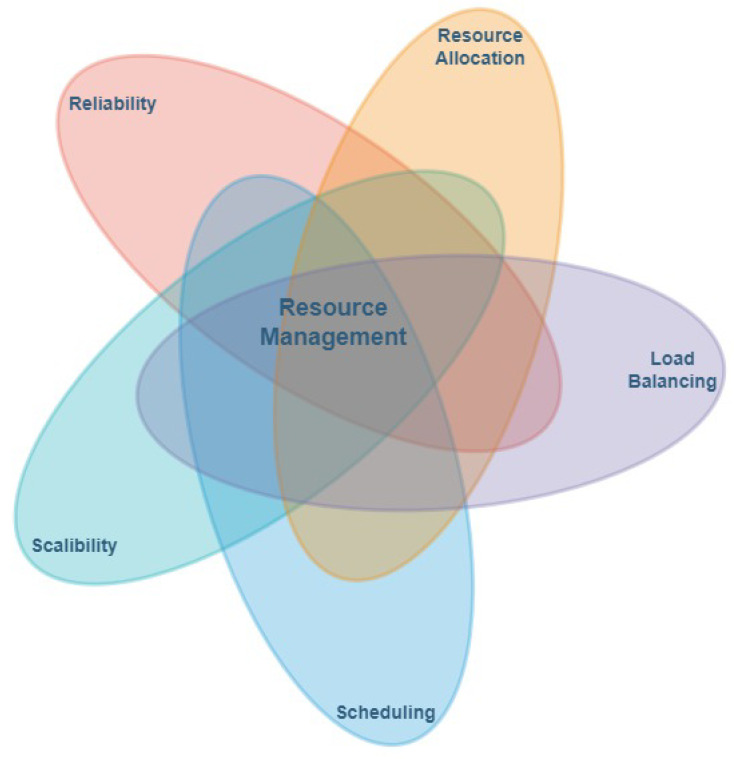
Resource management techniques.

A dependable and satisfying user experience fundamentally relies on the system’s ability to maintain consistent performance and service quality. This is achieved through stringent adherence to Quality of Service (QoS) requirements, which ensure timely task execution, minimal latency and uninterrupted service delivery. To meet these QoS objectives, operational costs must be optimized through intelligent resource utilization and energy-aware management strategies. In turn, such optimization contributes to higher computational efficiency by minimizing resource idleness, avoiding overload conditions and maximizing throughput. Consequently, the overarching principle of effective resource management serves as the foundation for achieving cost efficiency, performance optimization and sustainable reliability across distributed computing environments [30] as shown in Figure 2.

Efficient resource management continues to be a critical concern across various computing paradigms. In cloud environments, the centralized architecture often leads to substantial bandwidth consumption and network congestion, as large volumes of user-generated data must traverse long communication paths. This centralized dependency introduces considerable latency, rendering cloud systems less suitable for delay-sensitive real-time applications [31]. In contrast, fog computing alleviates such latency by bringing computation closer to the data sources. However, it introduces its own set of challenges. The decentralized and resource-constrained nature of fog nodes, characterized by limited processing power, memory and storage, makes effective resource orchestration more complex. Consequently, achieving secure, adaptive and efficient resource management within fog infrastructures is essential to ensure consistent performance and reliability under highly dynamic and unpredictable workloads [29].

Similarly, incorporating a security perspective into the resource management framework has been a crucial aspect of acquiring critical clinical data that needs protection during data acquisition, processing and transmission. Data protection mechanisms need to be employed across the three-tier architecture and evaluate their impact on system latency and energy consumption. Secure task offloading and secure transmission are particularly important in distributed fog architecture and need a verifiable match of the originating device. These security measures help to protect the critical healthcare data.

In this study, a meta-heuristic driven framework is introduced to enhance the efficiency of resource management and task scheduling within fog computing environments. Rather than focusing solely on static allocation policies, the proposed method employs adaptive optimization techniques to intelligently balance workloads and allocate computational resources in response to dynamic network conditions. The underlying goal is to achieve a more efficient utilization of fog infrastructure while indirectly reducing critical performance indicators such as makespan, execution cost and overall task completion time. By harmonizing these objectives, the proposed strategy contributes to improved system responsiveness, reduced operational overhead and sustained service quality across heterogeneous fog nodes.

## 5. Problem Formulation and Optimization Modeling

This work introduces a resource management framework tailored for latency-sensitive healthcare applications operating within fog-enabled environments. By exploiting the spatial closeness of fog nodes to end-user medical devices. The proposed architecture significantly minimizes communication delays and facilitates real-time processing of critical healthcare data. To make the system capable of operating and utilizing resources efficiently, the following conditions must be met:

- To minimize system key performance parameters in terms of response time, cost and execution.
- To maximize system resources and ensure system reliability.
- To meet the quality of service (QoS) constraints pertaining to healthcare applications.

### 5.1. Optimization Model

The proposed architectural framework model is assessed by using the optimization function. The optimization function is used for the performance of different performance parameters like makespan and cost execution of the performing nodes to show the proposed algorithm successfully performs at the given scenario.

(1) $$\mathrm{Min}\sum_{n=1}^{N}\left(Makespan\right)+\left(ExecutionCost\right)$$

s.t.

(2) $$CU_{r}+T_{load}\leq MU_{r},\forall n\in N_{k}$$

(3) $$UT_{i}^{m}\leq R_{m}$$

(4) $$K_{n,i}=0,1\leq i\leq T_{n}$$

Equation (1) formulates the joint optimization objective aimed at minimizing both execution time and computational cost. Equation (2) defines the feasibility constraint, ensuring that the cumulative load on a fog node, after incorporating new task requests, does not exceed its maximum resource capacity; only then is task execution permitted. Equation (3) introduces the optimization constraint associated with tasks that remain unscheduled within the system. Equation (4) characterizes the process of task offloading from end devices to fog nodes, representing the decision mechanism for distributed computation. Finally, Equation (5) delineates the binary offloading decision variable, where a value of 0 indicates that the task is offloaded to fog nodes for near-edge processing, while a value of 1 denotes that the task is allocated to cloud servers for high-complexity computation and long-term data management.

(5) $$t_{n,i}=\left\{\begin{array}{c} 0,|w|=f\\ 1,|w|=c \end{array}\right.$$

Here, $t_{n,1}$ = 0 denotes that the *i*th task is allocated to the available fog nodes for immediate and latency-sensitive execution. Conversely, $t_{n,1}$ = 1 signifies that the task is offloaded to the cloud layer, where it is processed for large-scale analytics and long-term storage purposes. In the proposed intelligent healthcare framework, the fog layer is prioritized as the primary computational tier, given its proximity to data sources and its ability to ensure low-latency processing for time-critical healthcare applications. The variable *w* denotes the type of computational node participating in the task execution process. The primary contribution of the proposed work mainly focused on performance-centric optimization for fog-enabled IoHT systems. The security considerations discussed in Section 4 highlight essential operational constraints that directly influence resource availability, task execution delay and execution cost. In particular, mechanisms such as encryption, authentication and secure data transmission introduce additional computational and network overhead that can affect the task scheduling and offloading dynamics modeled in our framework. Although these security factors are not explicitly embedded in the present mathematical formulation, they provide a foundation for extending the proposed MPSO-based optimization framework into a security-aware multi-objective model.

### 5.2. Proposed MPSO Modifications

We propose a Modified Particle Swarm Optimization (MPSO) algorithm for IoHT task scheduling in hierarchical fog environments. The modifications aim to minimize system makespan and execution cost while improving convergence rate as described in Equation (1).

#### 5.2.1. Customized Fitness Function

The fitness function jointly optimizes system makespan and execution cost and is given below.

(6) $$F_{i}=\alpha \frac{Makespan_{i}}{Makespan_{\max}}+\beta \frac{ExectionCost_{i}}{ExecutionCost_{\max}},$$

where $L_{i}$ and $E_{i}$ denote the makespan and execution cost of particle *i*, $L_{\max}$ and $E_{\max}$ are their respective maxima, and α+β=1 to balance the objectives.

#### 5.2.2. Adaptive Inertia Weight

A time-varying inertia weight balances exploration and exploitation with the equation given below.

(7) $$w\left(t\right)=w_{\max}-\frac{w_{\max}-w_{\min}}{T_{\max}}\cdot t,$$

where *t* is the iteration, $w_{\max}$/$w_{\min}$ are maximum/minimum weights, and $T_{\max}$ is the maximum iteration count.

#### 5.2.3. Modified Velocity and Position Updates

The velocity and position updates are modified as

(8) $$v_{i}\left(t+1\right)=w\left(t\right)v_{i}\left(t\right)+c_{1}r_{1}\left(p_{i}^{\mathrm{best}}-x_{i}\left(t\right)\right)+c_{2}r_{2}\left(g^{\mathrm{best}}-x_{i}\left(t\right)\right)+\gamma \Delta_{i},$$

(9) $$x_{i}\left(t+1\right)=x_{i}\left(t\right)+v_{i}\left(t+1\right),$$

with $c_{1}$, $c_{2}$ as acceleration coefficients, $r_{1},r_{2}∼U\left(0,1\right)$, and $\Delta_{i}=\eta \left(x_{\mathrm{rand}}-x_{i}\left(t\right)\right)$ adding controlled disturbance to avoid local optimum.

#### 5.2.4. Impact on Convergence

The proposed modifications improve convergence as follows:

1. Normalizing fitness to balance makespan and execution cost.
2. Adaptive inertia providing smooth transition from exploration to exploitation.
3. Velocity disturbance allows escape from local minima.

The novelty of the proposed MPSO lies in its problem-specific algorithmic design, which integrates IoHT contextual information directly into particle behavior, rather than relying on generic PSO parameter adjustments. In particular, MPSO employs a sensor-driven adaptive search mechanism in which inertia and velocity components dynamically respond to real-time variations in task heterogeneity and fog-node workload, enabling the search process to adapt to rapidly shifting IoHT conditions. Furthermore, the algorithm introduces a topology-aware multi-region exploration strategy that coordinates particle movements across heterogeneous fog nodes with differing capacities and communication delays, which is not present in conventional PSO variants. This is complemented by a context-sensitive perturbation scheme, where repulsion strength is modulated by local congestion levels and execution cost gradients, allowing more effective escape from local optima in nonlinear resource landscapes. These domain-oriented mechanisms collectively establish MPSO as a distinctive optimization framework tailored to fog-enabled IoHT environments, beyond incremental modifications of classical PSO.

### 5.3. Algorithmic Process

Algorithm 1 presents the efficient resource allocation and scheduling strategy, where tasks are offloaded to fog devices at multiple levels. The swarm optimization technique has two main characteristics: position, denoted as ($x_{1}$, $x_{2}$…$x_{n}$); and velocity, denoted as ($v_{1}$, $v_{2}$…$v_{n}$). In the process, each particle is represented as a fog node, which identifies the optimal solution using local and global vicinity, respectively. Therefore, the proposed algorithm consists of a multi-level task offloading strategy to appropriate fog devices with immediate response time.

**Figure 3:**
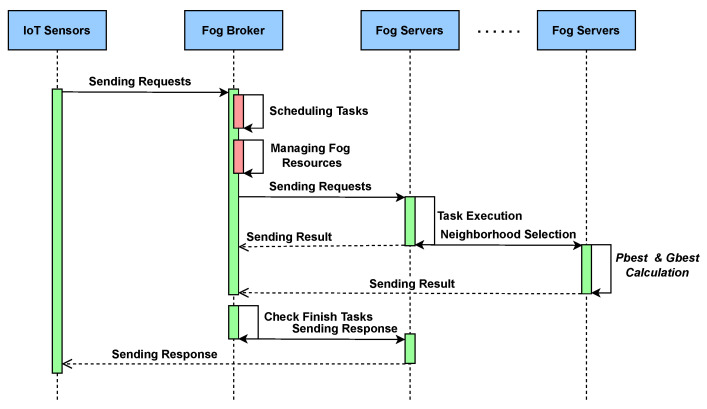
Sequence diagram of proposed MPSO.

Figure 3 shows the proposed algorithm for the three-tier framework. The IoT user sends requests to the fog broker, which manages the fog device resources as well as the requests coming from the IoT layer. The fog broker schedules the tasks to appropriate fog devices and ensures their timely execution. The fog broker monitors and controls the overall process continuously and checks the status of task completion. The results are sent back to the end users.

The complexity analysis of the proposed algorithm reveals that, in the best-case scenario, it exhibits θ(n) time complexity, as the algorithm performs in a linear fashion. This occurs when the condition in the outer loop is consistently false, resulting in minimal iterations. The outer loop execution performs the maximum number of iterations, leading to time complexity in the worst-case scenario.
**Algorithm 1** Enhanced Modified Particle Swarm Optimization (E-MPSO) for Intelligent Resource Allocation and Task Scheduling **Require:  **● Task set $T=\{T_{1},T_{2},\ldots ,T_{m}\}$  ●   Fog device set $F=\{F_{1},F_{2},\ldots ,F_{k}\}$  ●   Resource capacity $C_{r}$, utilization threshold $MU_{r}$  ●   Task requirements $R_{m}$, desired task MIPS $UT_{i}^{m}$ **Ensure:**  Optimal mapping of tasks to fog devices to minimize *makespan* and *execution cost*                                ▹**Stage 1: Initialization** 1: Initialize population of particles representing task-device mappings 2: Assign random positions and velocities to each particle 3: Set individual best positions $P_{best}$ and global best $G_{best}$ 4: Initialize resource utilization matrix U for all $F_{k}$                     ▹**Stage 2: Feasibility Evaluation** 5: **for** each task $T_{i}\in T$ **do** 6:     **for** each fog node $F_{j}\in F$ **do** 7:         **if** $\left(C_{r}\left(F_{j}\right)+R_{m}\left(T_{i}\right)\right)\leq MU_{r}\left(F_{j}\right)$ **then** 8:            Compute task execution time $E_{ij}=\frac{R_{m}\left(T_{i}\right)}{C_{r}\left(F_{j}\right)}$ 9:            Compute energy cost $EC_{ij}$ and communication delay $D_{ij}$ 10:            Evaluate fitness $f_{ij}=\omega_{1}E_{ij}+\omega_{2}EC_{ij}+\omega_{3}D_{ij}$ 11:         **else** 12:            Mark $F_{j}$ as overloaded 13:         **end if** 14:     **end for** 15: **end for**                        ▹**Stage 3: Particle Update** 16: **for** each particle *p* **do** 17:     Update velocity: $v_{p}\left(t+1\right)=\omega v_{p}\left(t\right)+c_{1}r_{1}\left(P_{best,p}-x_{p}\right)+c_{2}r_{2}\left(G_{best}-x_{p}\right)$ 18:     Update position: $x_{p}\left(t+1\right)=x_{p}\left(t\right)+v_{p}\left(t+1\right)$ 19:     Re-evaluate fitness for updated positions 20:     Update $P_{best,p}$ and $G_{best}$ based on fitness improvement 21: **end for**                ▹**Stage 4: Task Scheduling and Execution** 22: **for** each task $T_{i}$ **do** 23:     Select fog node $F_{opt}$ with minimal fitness value 24:     Allocate $T_{i}\to F_{opt}$ 25:     Execute $T_{i}$ and update utilization $C_{r}\left(F_{opt}\right)$ 26: **end for**

## 6. Configuration and System Setup Details

To make the framework model effective, the following configurations and system setups have been made to obtain the acquired results.

### Configuration Setup

To perform the experimental operations using the iFogSim toolkit, which is primarily concerned with modeling and simulation of resource management techniques. The accuracy and the reliability of the obtained results are associated with the strategies defined in the simulation parameters defined in Table 1. This article proposed a well-established simulation environment to perform the proposed algorithm and obtained the desired results in terms of makespan and execution cost in computationally constrained conditions. The experimental setup details for performing the proposed algorithm are outlined in Table 2.

## 7. Result and Discussion

### 7.1. Analysis of Time Execution

**Figure 4:**
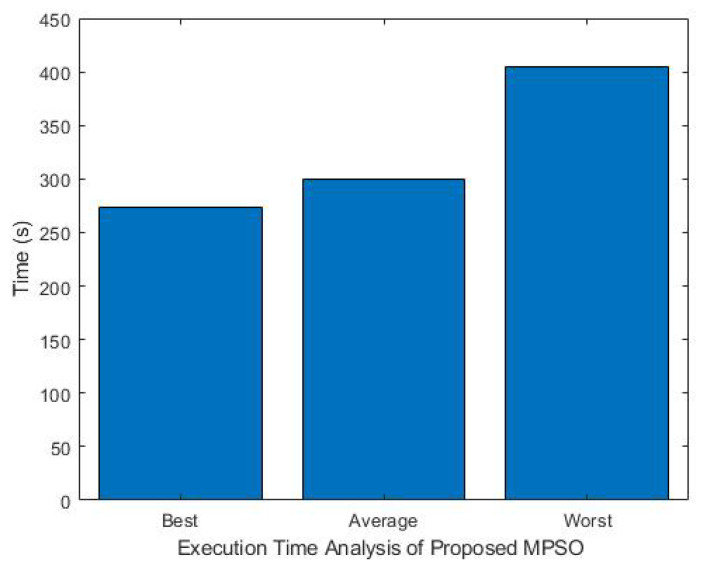
Execution time analysis of proposed MPSO algorithm.

Figure 4 shows the execution time performance of the MPSO algorithm across various fog device setups and task volumes. Optimal scenarios with smooth convergence lead to significantly reduced execution times, reflecting effective resource-task alignment. Conversely, improper resource allocation results in longer execution times. The obtained results derived from extensive simulations and testing of system nodes and task execution time to measure the system speed and stability due to the efficient resource allocation strategy.

### 7.2. Analysis of Makespan

The system’s crucial performance parameter, i.e., system makespan, is continuously observed after several simulation runs, and its performance stability is carefully checked. The obtained results demonstrate that the performance of the proposed technique is significantly better compared to other SOTA algorithms. These novel results for real-time applications were due to efficient resource allocation and task scheduling strategy and are largely attributable to the introduction of a multi-level task execution strategy, which distinguishes the algorithm from its counterparts. Furthermore, the algorithm optimally exploits the available fog computing resources, thereby effectuating a substantial reduction in the total makespan as depicted in Table 3.

**Figure 5:**
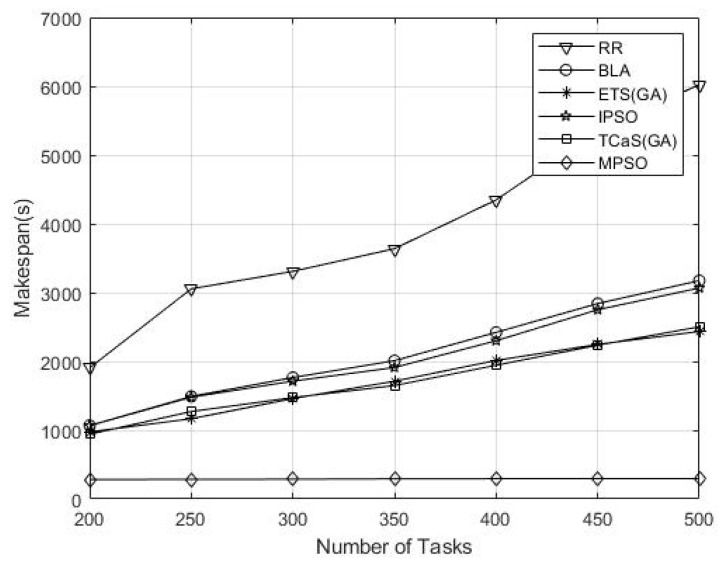
Performance result of the makespan (s).

Figure 5 demonstrates that the proposed technique was executed for a number of iterations and obtained the results, which indicates that the proposed algorithm outperformed others due to its low computational time and fast convergence rate. Furthermore, the results indicate that the proposed technique maintains stable performance even when fog nodes exhibit varying resource capacities in heterogeneous conditions. While in homogeneous conditions, all fog nodes have identical computing and communication capabilities. The above results show a significant reduction in makespan analysis in a heterogeneous environment due to an adaptive particle update mechanism. The adaptive particle update enables efficient task node matching even when resource disparity exists. The results signify and confirm the robustness and applicability of MPSO in diverse IoHT heterogeneous environments.

### 7.3. Analysis of Execution Cost

The execution cost measured the system computational performance per unit cost and is usually represented as unit grid dollar (G$). A higher execution cost indicates increased system overhead, whereas low cost values reflect better system performance as shown in Table 4.

**Figure 6:**
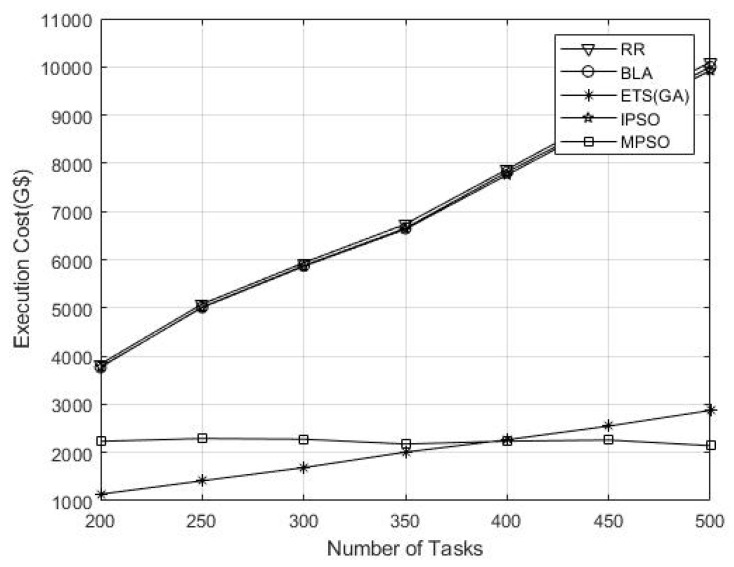
Performance result of the execution cost (G$).

The proposed technique performed better compared to other techniques for the number of tasks 400 and above. This is due to the stability of the algorithm and its fast convergence rate, as shown in Figure 6.

### 7.4. Analysis of Mixed Healthcare Workloads

**Figure 7:**
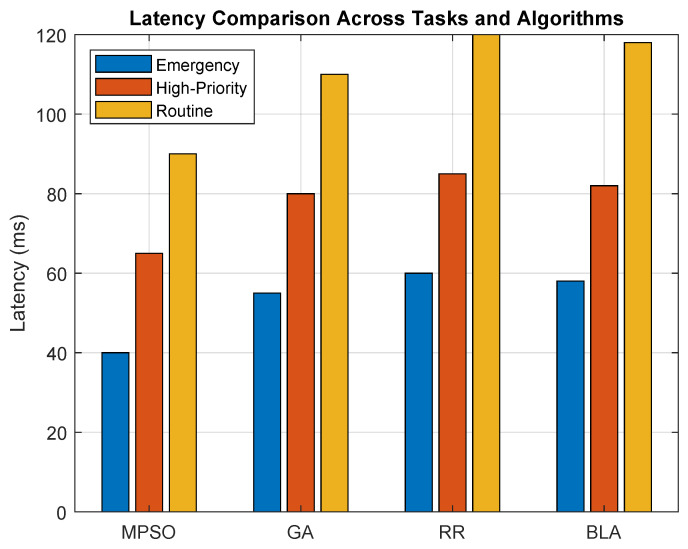
Performance analysis of mixed healthcare workloads of various algorithms.

Figure 7 demonstrates that the proposed MPSO scheduling technique provides a scalable and robust solution for delay-sensitive healthcare workloads in fog-enabled IoHT environments. Analyzing consistently the lower latency across various workloads, such as emergency tasks, high-priority tasks and routine tasks, substantially reduces the response time, especially noticed in emergency workloads. Further, our results confirm that MPSO is well suited for real-world healthcare systems for timely processing due to its sensitivity. The algorithm’s superior performance under heterogeneous nodes, mixed workloads and dynamic task conditions validates its adaptability and highlights its ability to optimize resource utilization without compromising service quality. These outcomes support our conclusion that MPSO offers a reliable, responsive and high-performance strategy for intelligent resource management techniques.

**Figure 8:**
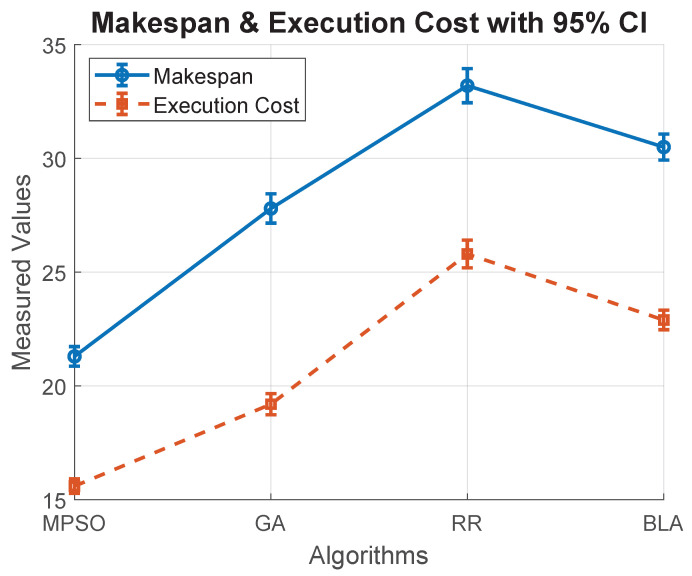
Makespan and execution cost confidence intervals of various algorithms.

Figure 8 illustrates the comparison of four scheduling algorithms to analyze the performance in terms of makespan and execution cost. The performance of each was evaluated over 30 independent simulation runs, with both metrics showing a 95% confidence interval rate, thus ensuring statistical reliability and highlighting the variability in outcomes. The figure also demonstrates the relative efficiency of the algorithms, where lower values indicate better scheduling performance. This integrated visualization enables quick comparison of computational delay (makespan) and resource utilization (execution cost).

### 7.5. Performance Comparison

A comparative analysis of the proposed framework was conducted against a selection of recently published studies, as summarized in Table 5. This comparison is based on assessing the key performance parameters in terms of makespan, execution time and cost of execution for the overall system effectiveness to determine if the proposed technique meets the required solutions as compared to other existing techniques mentioned in various literature presented in the table. The proposed tri-tier architectural framework is evaluated using real-time sensor data for measuring the effect of system performance parameters, and it is evident from the obtained results that the proposed technique outperformed other meta-heuristic techniques.

It is worth mentioning to note that several recent DRL- and GNN-based task offloading frameworks are discussed in the related work; these methods were not incorporated as experimental baselines in this study. The primary reason is that our evaluation environment is built on iFogSim, which natively supports deterministic and metaheuristic scheduling models but does not provide stable or reproducible implementations of DRL-based schedulers. Integrating architectures such as ReinFog or DQN-based task offloading would require substantial modification of the simulator, including custom environment agent interaction loops, online learning modules and action abstractions, which falls outside the technical scope of this optimization-oriented metaheuristic framework. For this reason, GA, IPSO, BLA and RR were selected as representative baselines within the same algorithmic family for comparison purposes as presented in Table 3 and Table 4.

## 8. Conclusions

Fog computing provides resources near to the edge users without any delay. The execution of IoT tasks to fog devices is a sustainable and effective way of fog-IoT connection. However, unlike the cloud, which offers centralized data centers with abundant computational power and storage, fog operates with limited processing power and storage capacity closer to the network edge. But due to the centralized and geographically wide distribution of servers, there is a communication latency. Therefore, fog provides a low cost. low response time, reliable and better quality of services for real-time applications. To address the challenges posed by unpredictable and dynamic service requests, fog computing requires a robust resource management strategy that can efficiently utilize limited resources while minimizing latency. This article proposes an effective resource allocation and task scheduling approach based on a Modified Particle Swarm Optimization (MPSO) algorithm. The primary objective of employing MPSO is to minimize execution time, reduce makespan, and lower the overall execution cost, thereby enhancing the performance of delay-sensitive applications in fog environments.

In the future, advanced machine learning techniques for intelligent IoHT task scheduling will be explored. Deep reinforcement learning (DRL) and DQN can enable real-time dynamic task offloading, while graph neural networks can model complex device task dependencies to enhance system efficiency, reliability, robustness and resilience. Moreover, security overhead functions, trust management and adversarial resilience metrics into both the objective formulation and algorithmic design can be a future prospect to incorporate, thus enabling a unified security performance optimization strategy for next-generation fog-enabled IoHT environments.

## Figures and Tables

**Table 1:** Simulation configuration parameters.

Category	Parameter Description and Values
**Infrastructure Setup**	Cloud Server: 1 (max)
	Fog Devices: 100–500
	IoHT Sensors: 50–100
	IoHT Tasks: 500–1000
**Computational Resources**	Fog Device Computation Capacity: 5600 MIPS
	Fog Storage Capacity: 4000 MB
	Cloud Storage Capacity: 40,000 MB
**Network Configuration**	Latency (Sensor → Edge): 1–2 ms
	Latency (Edge → Fog Layer 2): 3–5 ms
	Latency (Fog Layer 2 → Fog Layer 1): 8–12 ms
	Latency (Fog Layer 1 → Cloud): 80–100 ms
	Bandwidth (Sensor Links): 1–5 Mbps
	Bandwidth (Edge/Fog Links): 50–200 Mbps
	Bandwidth (Fog–Cloud): 1000 Mbps
**Power Consumption**	Cloud Server Power (Busy/Idle): 1648 W/1332 W
	Fog Device Power (Busy/Idle): 107 W/83 W
**IoHT Task Characteristics**	Instruction Length: 500–5000 MIPS
	Input Data Size: 10–500 KB
	Output Data Size: 3–100 KB
	Task Deadlines: 80–500 ms
	Task Types: ECG, SpO_2_, BP, temperature, emergency alerts
**MPSO Parameters**	MPSO Population Size: 30
	MPSO Maximum Iterations: 100
	Cognitive Coefficient $c_{1}$: 1.5
	Social Coefficient $c_{2}$: 1.5
	Inertia Weight $w_{\max}/w_{\min}$: 0.9/0.4
	Disturbance Factor γ: 0.05
	Learning Rate η: 0.1
	Fitness Weighting α/β: 0.6/0.4
**Dataset Description**	Data Source: UTeM Clinical Healthcare Dataset (ECG-based)
	Data Type: real-time physiological and demographic records
	Physiological Parameters: heart rate, ECG waveforms, vital signs
	Demographic Parameters: age, gender, height and weight
	Record Format: structured patient entries with time stamped ECG data
	Dataset Usage: evaluation of makespan and execution cost under MPSO
	Privacy Status: restricted
	Integration Framework: 3-tier cloud-fog-IoT architecture

**Table 2:** System setup details.

Hardware/Software	Details
Toolkit	iFogSim
Toolkit Version	3.0.3
Editor	Eclipse 4.14
Programming Environment	Java
Runtime Environment	JRE 13.0.1 version
System	Intel Core 1.83 GHz
Operating System	Windows 10 (Professional)
RAM	8 GB

**Table 3:** Makespan analysis of different algorithms.

Task Numbers	RR	BLA	IPSO	TCaS (GA)	ETS (GA)	Proposed MPSO
200	1898.2	1067.49	1065.94	940.87	974.89	283.79
250	3054.8	1490.65	1479.84	1270.96	1166.40	284.95
300	3309.14	1765.49	1712.76	1473.79	1460.07	287.76
350	3638.19	2010.74	1911.27	1649.04	1712.17	293.08
400	4347.64	2421.98	2300.51	1944.58	2014.12	294.83
450	5350.35	2840.79	2751.29	2235.16	2247.18	294.96
500	6023.74	3174.39	3067.26	2503.09	2435.12	295.98

**Table 4:** Execution cost analysis of different algorithms.

Task Numbers	RR	BLA	IPSO	TCaS (GA)	ETS (GA)	Proposed MPSO
200	3926.3	3688.46	3862.19	3844.35	1346.12	2232.32
250	4234.17	4261.55	4758.74	4988.94	1413.19	2290.44
300	5935.94	5877.41	5862.52	5832.69	1686.92	2275.58
350	6738.19	6653.37	6632.38	6607.52	2007.76	2176.86
400	7875.39	7816.04	7759.95	7738.56	2265.63	2234.93
450	9016.59	8926.57	8876.35	8845.90	2548.51	2259.08
500	10,097.75	9995.97	9921.76	9902.64	2872.86	2141.62

**Table 5:** Performance comparison of the proposed work with existing literature.

Ref.	Framework	Domain	Sensors	Data Type	Parameters	Main Contribution
[13]	Cloud–Fog–IoT	Healthcare	Heartbeat, blood sugar	Synthetic	Delay, energy, network usage	Dynamic workload-aware scheduling for healthcare.
[16]	Cloud–Fog–IoT	Healthcare	ECG, EEG	Synthetic	Latency, network utilization, energy	Shortest job-first scheduling for patient monitoring.
[32]	Cloud–Fog–IoT	Healthcare	ECG, EMG, EEG	Synthetic	Power, execution time, network usage	Predicts accuracy and QoS for cardiac disease analysis.
[33]	Fog–IoT	Healthcare	ECG, PPG	Synthetic	Sensitivity, precision, accuracy	Temporal sensitivity analysis for pet healthcare.
[34]	Cloud–Fog–IoT	Healthcare	Blood pressure	Real-time	Delay, energy	Multi-agent fog system for healthcare task coordination.
[35]	Cloud–Fog–IoT	Healthcare	Biosensors	Synthetic	Latency, execution time, detection accuracy	Tri-fog health architecture for wearable monitoring.
[36]	Cloud–Fog–IoT	Healthcare	ECG, EEG	Real-time	Accuracy, sensitivity, specificity	CNN-based cancer detection framework.
[37]	Fog–IoT	Healthcare	ECG	Real-time	Allocation cost, response time	Reinforcement learning for dynamic resource allocation.
**Prop.**	Cloud–Fog–IoT	Healthcare	ECG, EEG	Real-time	Execution time, makespan, cost	Optimized resource utilization to enhance system performance.

## Data Availability

No new data were created or analyzed in this study. Data sharing is not applicable to this article.
